# Spatial analytics to elucidate the incubation period and drivers of visceral leishmaniasis: case of Turkana County in Kenya

**DOI:** 10.3389/fdgth.2025.1643314

**Published:** 2025-10-10

**Authors:** Kennedy Senagi, Maureen Nzilani, Evans Omondi, David P. Tchouassi, Tobias Landmann, Damaris Matoke-Muhia, Emmanuel Okunga, Barrington Gesimba, Elfatih M. Abdel-Rahman, Dawn Maranga, Joseph M. Ndungu, Daniel Masiga

**Affiliations:** ^1^ International Centre of Insect Physiology and Ecology, Nairobi, Kenya; ^2^ Institute of Mathematical Sciences, Strathmore University, Nairobi, Kenya; ^3^ African Population and Health Research Center, Nairobi, Kenya; ^4^ Kenya Medical Research Institute, Nairobi, Kenya; ^5^ Ministry of Health, Nairobi, Kenya; ^6^ Foundation for Innovative New Diagnostics, Geneva, Switzerland

**Keywords:** disease modelling, data science, epidemiology, disease risk, environmental influences

## Abstract

**Introduction:**

Visceral leishmaniasis (VL) is a severe and neglected tropical disease of public health concern. VL is fatal if not treated. Kenya has experienced multiple outbreaks of the disease since 2017. The underlying drivers of the disease risk dynamics, as well as the incubation period, are not well understood.

**Methods:**

We implemented statistical (spatial logistic regression and Bayesian spatial) and machine learning (random forest, support vector machine, AdaBoost, logistic regression, and extra trees) models to estimate the incubation period and predict areas of low/high risk in Turkana County, an endemic VL foci in Kenya. Two-year (2019–2020) patient data were sourced from 12 VL treatment centers in Turkana County. Environmental and weather data were sourced from satellites, while demographic data were extracted from the Kenyan Population and Housing Census 2019 dataset. The environmental and weather data were lagged up to 8 months to mimic the disease incubation period.

**Results:**

The AdaBoost was the best-performing classifier with an area under the curve of the receiver operating characteristic value of 71.2%. The model predicted three months as the optimal incubation period. Age, distance to a healthcare facility, mean monthly humidity, greenness, and total precipitation were identified as the five main predictors. The epidemiological risk map (for December 2024) was generated and deployed on the Web (https://dudumapper.icipe.org/). The Kerio Delta, Lokori, and the shores of the Lake Turkana regions were predicted to have a mid to high risk/number of cases.

**Discussion:**

These data-driven findings can improve the understanding of VL risk dynamics and support decision makers in the preparation, mitigation, and elimination of VL.

## 1 Introduction

Leishmaniases are a group of diseases caused by protozoan parasites of the genus *Leishmania*, transmitted by sandflies. Leishmaniases affect humans. The three main forms of leishmaniasis are visceral, cutaneous, and mucocutaneous. Visceral leishmaniasis (VL), known as kala-azar, is the most severe form of leishmaniasis and is fatal if left untreated. The bites of an infected female phlebotomine sandfly infect humans with the *Leishmania donovani* or *Leishmania infantum* parasites, which cause VL. *Leishmania donovani* is the main causative parasite species of VL, especially in Sub-Saharan Africa, while *Leishmania infantum* is common in the Americas. VL is fatal if it is not treated in time. VL is endemic in about 80 countries around the world. It is estimated that 50,000 to 90,000 new VL cases are reported annually (1, 2).

In 2022, approximately 66% of the global cases of VL were reported from the eastern African corridor, and the disease is targeted for elimination by 2030 (3). Approximately half or more of these cases were children under 15 years of age. However, globally, up to 45% of VL cases are estimated to remain unreported, probably due to the epidemiological and clinical diversity of the disease, which poses significant challenges for surveillance, diagnosis, and treatment. In humans, VL is characterized by weight loss, irregular bouts of fever, anemia, and enlarged spleen and liver (2, 4–6).

VL is a climate-sensitive disease in that changes in weather patterns (mainly temperature, rainfall, and relative humidity) influence the geographic distribution and population of phlebotomine sandflies (7–11). Specifically, temperature affects the developmental cycle of *Leishmania* promastigotes in sandflies and the life cycle of vectors. Consequently, warmer climates alter the distribution, survival, population size, and competency of phlebotomine sandflies. Lower rainfall increases the probability of invasion (2). In addition, environmental (such as the presence of vegetation (11–13), type of soils (13, 14), and presence of ant-hill mounds (15, 16)), demographic [such as population density (17, 18), and age structure (19, 20)], socio-economic (such as types of houses (11, 16)), and many other factors, contribute to the transmission and (in/re)surgency of VL. The presence of VL hosts (humans or animals), termite hills, acacia trees, and water bodies is a risk factor as they create suitable habitats for the feeding, breeding, and resting for vectors (21–23). However, the interaction and impact of these variables on VL’s geographical transmission are not fully understood.

Based on accumulated cases (from 2007 to 2022) and meteorological data, (24) explored the applications of machine learning models to predict leishmaniasis outbreaks in selected cities in Brazil. The machine learning models were evaluated by the root mean squared error, showing the potential of the models in predicting leishmaniasis outbreaks. In Western and Central China, from 2007 to 2017, data collection, (25), analyzed the spatiotemporal patterns of annual human VL cases using the boosted regression tree model and spatial correlation techniques. This gave a better understanding of the spatial risk factors driving the spread of VL and identified potential endemic risk regions. Kumar et al. (26) configured the support vector regressor with the radial basis function kernel to assess the impact of climate change on disease outbreaks in Bihar, India. The model effectively identified temperature, wind speed, rainfall, and population density as significant contributors to VL risk.

In Kenya, 11 of the 47 counties are endemic to VL, which is approximately 62% of the total land area of the country. These counties are disproportionately poor, marginalized, (semi-)arid, and undeserved (27). In the country, VL cases are increasing and outbreaks have become recurrent in 2008, 2019, and 2025. New foci of the disease have been reported (e.g., Tharaka Nithi County), with sporadic cases reported in areas such as Kitui, Kajiado, and Marsabit counties, indicating an expanding geographical spread. There could be other new foci and cases that are unknown due to poor surveillance, diagnosis, treatment, and knowledge of drivers (which are mainly ecological and environmental). In the middle of the effects of climate change, new foci have been established and the increased incidence of VL infections reported, especially in arid and semi-arid areas of Kenya (such as Kajiado, Turkana, Marsabit, West Pokot, Isiolo, Garissa, Mandera, and Baringo). The control of the disease is mainly reactionary and targets humans already affected by the disease and seeking treatment in hospitals. There are hardly any epidemiological predictive models to inform various stakeholders about potential areas of high/low risk of the disease to targeted interventions (such as diagnosis and treatment, vector surveillance, integration of vector control mechanisms (e.g., pesticides), etc.). Neither is the optimal incubation period of the disease well known, since the infection to the onset of symptoms spans between a few weeks and 9 months. Turkana County is known to be one of the traditional and endemic sites of the disease (4, 27, 28). Taking into account epidemiological, environmental, and weather data from this county, this research developed statistical and machine learning models that unraveled the possible optimal incubation period (optimal time) in the future when we could anticipate the surge (increase) in human VL cases and the respective drivers. This information could be vital for various stakeholders (such as the Kenya Ministry of Health) in managing and controlling the disease.

## 2 Materials and methods

### 2.1 Study site

The study was carried out in Turkana County, which is the second largest of the 47 counties in the Republic of Kenya (29). Turkana County has an area of 71,597.6 km^2^ and represents 13.5% of the total land area in Kenya (30–32). Turkana County lies between $10^{\circ }$ 30’N and $50^{\circ }$ 30’N latitudes and $34^{\circ }$ 30’E and $36^{\circ }$ 40’E longitudes. The county is located in the Northwest of Kenya and borders Uganda to the west, South Sudan to the north, and Ethiopia to the northeast. The counties bordering Turkana County are West Pokot in the southwest, Baringo in the south, Samburu in the southeast, and Marsabit in the east (32). The vast eastern African Rift Valley traverses Turkana County. The county’s topography consists of low-lying plains and isolated hills and mountain ranges. The altitude extends from 369 m in Lake Turkana in the east to the highest point at around 900 m near the Ugandan border in the west (33). Turkana has a hot and dry climate with an annual temperature range between $20{\;}^{\circ }$C and $41{\;}^{\circ }$C, and a mean annual temperature of $30.5{\;}^{\circ }$C. Rainfall in the area is bimodal and highly variable. Long rains occur between April and July, and short rains occur between October and November. The annual rainfall is low, ranging between 52 and 480 mm with a mean rainfall of 200 mm (34). Rain patterns and distributions are unpredictable and unreliable. The county is prone to drought. Eighty percent of the county is classified as arid or very arid (33).

### 2.2 Data collection

The raw data consisted of patient, weather, environment, and demographic variables. Patient data were collected from 12 public hospitals that offered VL diagnosis and treatment services in Turkana County between 2019 and 2020. The data had a total of 1,673 records; positive and negative cases were 770 and 903, respectively. Patients’ data contained age, sex, patient village geo-coordinates, hospital name, date the patient was seen in the hospital by a physician, and VL test (determined by the rK39 rapid diagnostic test (RDT) or direct agglutination test (DAT) test kits) status variables. Furthermore, we obtained monthly weather data on temperature (minimum, maximum, and mean), average humidity, and average total precipitation from OpenMeteo (35) and EnviDat (36). We also used environmental data, with a 20 m satellite resolution, including derived tasseled cap vegetation index (wetness, greenness, and brightness), water bodies, land use and land cover (LULC), and soil type variables from the National Aeronautics and Space Administration (37). Demographic data had population density, which was obtained from the Kenyan National Bureau of Statistics (Population and Housing Census 2019) (38). Weather, environment, and demographic data were collected in reference to the villages of the patients and were dated 8 months before the date the patients were seen in the hospital; this was to align with the incubation period of VL that can last from a few weeks up to 9 months (39, 40).

### 2.3 Data pre-processing

**Figure 1:**
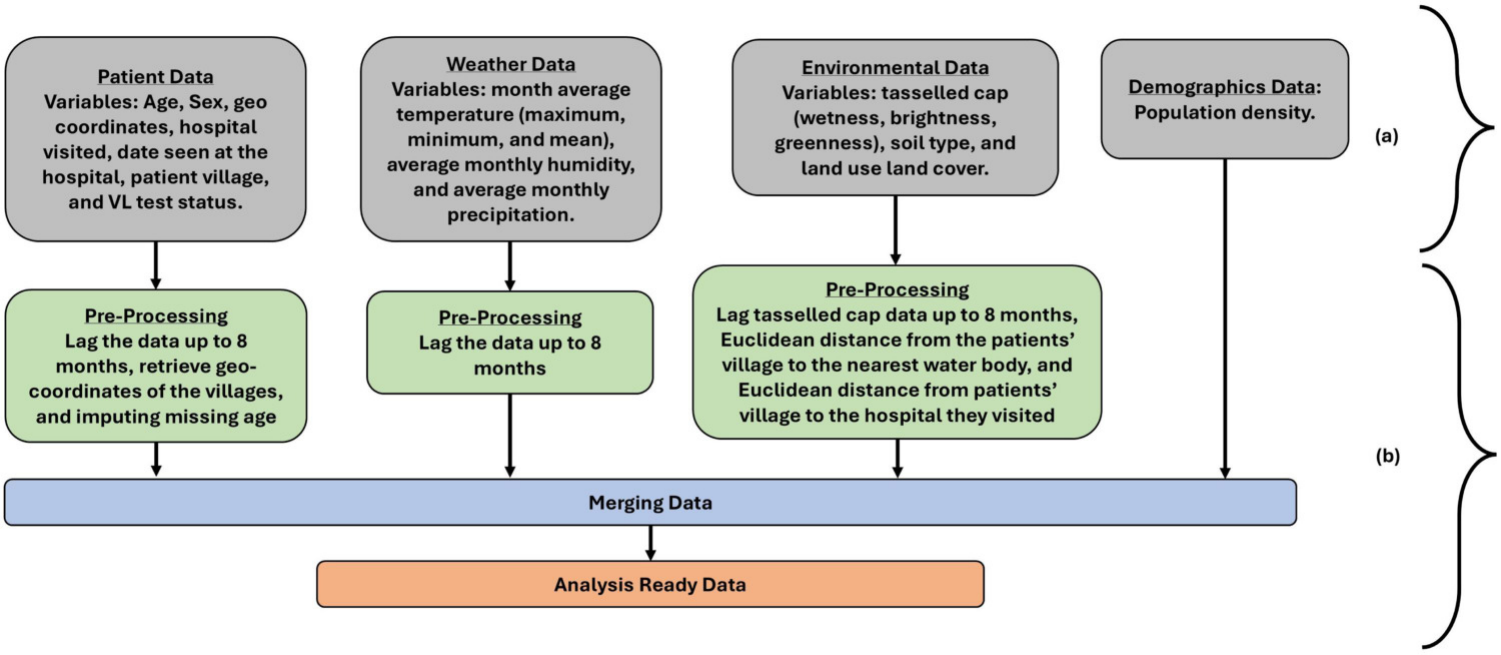
Steps involved in **(a)** raw data collection of respective variables and **(b)** data pre-processing. At the end of the data pre-processing step, the data was analysis-ready.

Figure 1 outlines the variables studied and the respective data pre-processing steps. The 8-month-lagged hospital, weather, and environmental data were stored in different comma-separated values (CSV) files. The geographical coordinates (latitude and longitude) of the village of patients and the VL treatment hospitals were identified and integrated into the dataset. In the hospital, the missing age was imputed using the mean age. We computed the Euclidean distance between (a) the patient village and the hospital visited, and (b) the patient village and the water bodies. These new distance variables were appended to the dataset. The hospital, weather, environment, and demographic data were augmented into 8 different CSV lagged files. The set of variables is listed and described in Table 1.

**Table 1:** A list and description of analysis data variables.

Item	Variable	Description
1	Gender	The sex (male or female) of the patient.
2	Age	Age of the individual
3	Latitude	Geographic latitude coordinate
4	Longitude	Geographic longitude coordinate
5	Distance to water bodies	Euclidean distance from the patients’ villages to the nearest water source in kilometers (km)
6	Population density	Number of people per square km
7	Mean temperature	Average monthly temperature in degrees Celsius
8	Minimum temperature	Average monthly lowest recorded temperature in degrees Celsius
9	Maximum temperature	Average monthly highest recorded temperature in degrees Celsius
10	Mean humidity	Average monthly humidity percentage%
11	Total monthly precipitation	Average monthly total rainfall in millimeters
12	Distance to healthcare	Euclidean distance from the patients’ villages to the nearest healthcare facility in km
13	Elevation	Height above sea level in meters
14	Soil type	Classification of soil at each patient village
15	Land use land cover (LULC)	Classification of a geographic area based on human activities, physical and natural features
16	Tasseled cap	Constitutes of greenness (which measures vegetation health and density), brightness (which measures the reflectance of the soil), and wetness (which measures moisture content in vegetation and soil)
17	Forest height	The average forest’s vertical structures at a radius of 5 km from the patients’ village
18	VL test results	Results of Rapid Diagnostic Test (RDT) using the rk39 antigen or Direct Agglutination Test (DAT). These are tests that detect whether the patient was infected with VL or not.

**Figure 2:**
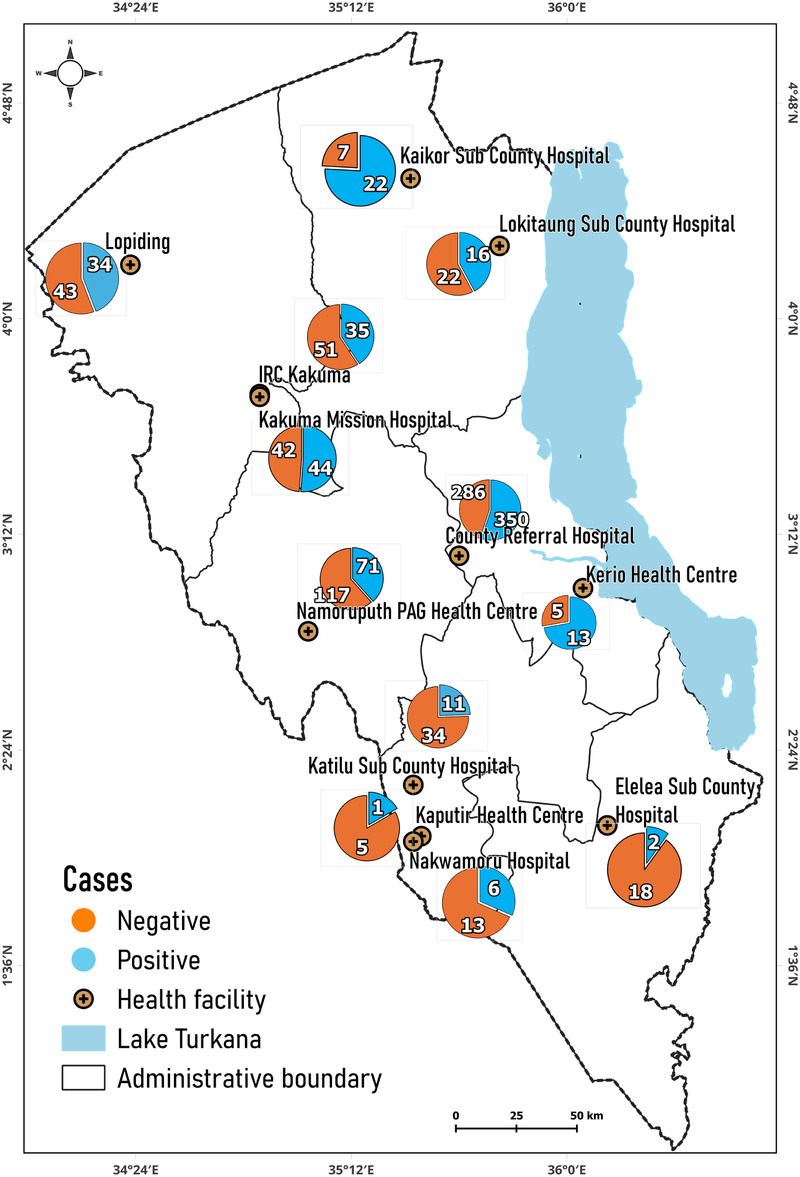
Distribution of positive and negative cases in hospitals. Map from: https://dudumapper.icipe.org/, licensed under MIT License.

The distribution of positive and negative VL cases in the different hospitals in Turkana County is illustrated Figure 2. The majority of positive VL cases were reported at the County Referral Hospital (350 cases), followed by the Namoruputh PAG Health Center (71 cases), Kakuma Mission Hospital (44 cases), and the International Rescue Committee (IRC) Hospital (35 cases). The number of negative cases of VL was the most frequent in the Turkana County Referral Hospital (266 cases), the Namoruputh PAG Health Center (114 cases), IRC Hospital (51 cases), and the Loping Sub-County Hospital (43 cases).

**Figure 3:**
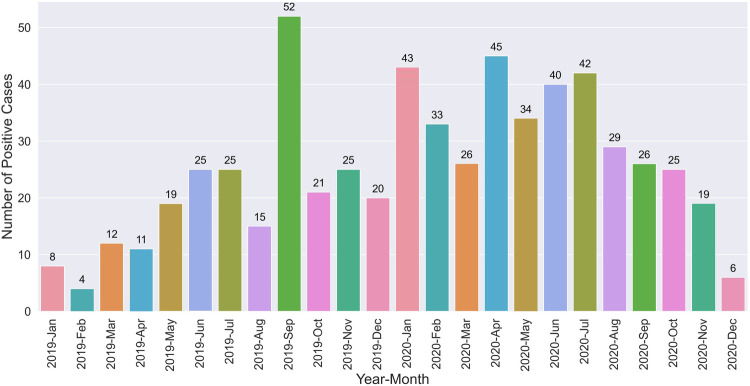
The count of VL positive cases across different months.

The distribution of positive VL cases over different months throughout the study period is shown in Figure 3. The trend shows variation in monthly case reports, with the highest 3-month peaks observed in September 2019 (52 cases), April 2020 (45 cases), and January 2020 (43 cases). The 3 months with low VL case numbers were in February 2019 (4 cases), December 2020 (6 cases), and January 2019 (8 cases).

### 2.4 Modelling

**Figure 4:**
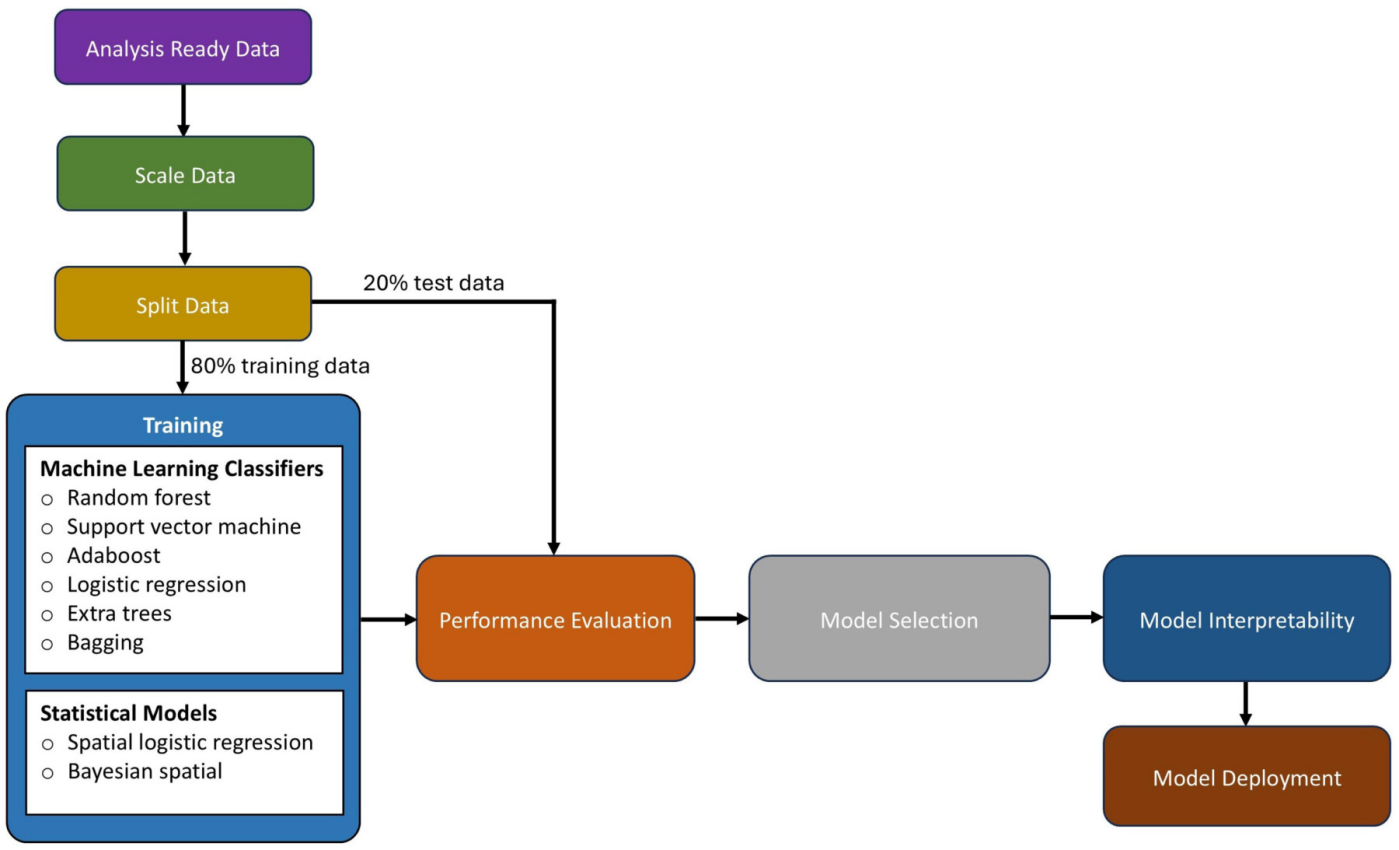
A workflow showing the data preparation and learning processes.

Figure 4 explains the phases of model implementation for both machine learning and statistical models. During the implementation process, the pre-processed dataset (from Section 2.3) was scaled using the Z-score, stated in Equation 1. In the equation, *z*, *x*, μ, and *s* denote the Z-score, the raw score, the mean of the data, and the standard deviation of the data, respectively. After scaling the data, it was randomized and split into train (80%) and test sets (20%). Splitting the data allowed the models to learn from a representative portion (train set) of the data, while the test set (unseen) was used for performance assessment.

(1) $$z=\frac{x-\mu }{s}$$

#### 2.4.1 Machine learning modelling

The machine algorithms can determine the most important and relevant drivers/variables (41–45). Considering the 8-month lagged dataset, we implemented several machine learning classification algorithms to identify the optimal lag and predict other possible areas where VL could occur. The machine learning algorithms were RF, AdaBoost, DT, SVM, logistic regression, and extra trees. The Sklearn (46) library in Python was used to implement machine learning algorithms. We note that the geo-location (latitude and longitude) was not used in training the algorithms, but the prediction results were associated with the geo-location and mapped.

i. *Random forest classifier:* The RF classifier is a machine learning algorithm that uses the bagging approach to generate multiple decision trees. The class voted for by the majority of each tree is taken as the final predicted class (47). The probability of the trees making the final prediction is represented in Equation 2. In the equation, y represents the label of the class for which the probability is estimated, T is the total number of trees, and $P_{i}(y)$ denotes the probability assigned to the class y by the $i^{th}$ tree.
  (2) $$RF(y)=\frac{1}{T}\sum_{i=1}^{T}P_{i}(y)$$
ii. *Support vector machine classifier:* The SVM classifier is a supervised machine learning algorithm that identifies the optimal hyperplane in a high-dimensional space to separate the classes in a dataset (48). When training a dataset, new points are classified based on their positions relative to the hyperplane (49). The SVM decision function is described in Equation 3. In the equation, $\alpha_{i}$ are the coefficients (Lagrange multipliers), $y_{i}$ are the class labels, $K(x,x_{i})$ is the kernel function, x is the test data point, $x_{i}$ is any support vector, and b is the bias term.
  (3) $$SVM(x)=\text{sign}\left(\sum_{i=1}^{n}\alpha_{i}y_{i}K(x,x_{i})+b\right)$$
iii. *AdaBoost classifier:* AdaBoost is a supervised machine learning algorithm that works by combining multiple weak classifiers into a stronger classifier. Initially, the algorithm assigns all data points the same weight. A weak classifier is trained on the dataset, and its errors are identified. Misclassified points are assigned higher weights to give them more importance in the next iteration. The process is repeated with multiple weak classifiers, and their outputs are combined by weighted voting to create a strong final model (50). Mathematically, AdaBoost is represented as Equations 4, 5 where $\alpha_{t}$ is the weight assigned to the classifier t, E is the error rate of the weak classifier, $h_{t}(x)$ is the output of the weak classifier t for input x.
  (4) $$\alpha_{t}=0.5\ln\left(\frac{1-E}{E}\right)$$
  (5) $$H(x)=\text{sign}\left(\sum_{t=1}^{T}\alpha_{t}h_{t}(x)\right)$$
iv. *Logistic regression:* Logistic regression is a supervised classification learning algorithm that transforms the response variables into a probability using the sigmoid function and converts the target/outcome variable into 0 or 1. Logistic regression estimates how a change in an independent variable affects the log odds of the predicted class, holding other variables constant. Ultimately, this model finds an optimal set of weights by minimizing the negative log-likelihood (51). Mathematically, logistic regression is expressed as Equation 6. In Equation 6, π(x) is the probability that Y=1 given X, $\ln\left(\frac{\pi (x)}{1-\pi (x)}\right)$ is the logit function that transforms the probability into an unbounded value.
  (6) $$\text{logit}(\pi (x))=\ln\left(\frac{\pi (x)}{1-\pi (x)}\right)=\beta_{0}+\beta_{1}x_{1}+\cdots +\beta_{p}x_{p}$$
v. *Extra trees:* The extra trees is a supervised learning algorithm. Like RF, this algorithm predicts by combining decisions from multiple trees. Extra trees work by generating random splits without looking for an optimal threshold, which the random forest does with bootstrapping sampling. This algorithm uses the entire dataset for each tree and then selects random splits to maintain randomization (52).

#### 2.4.2 Statistical modelling

*Spatial autocorrelation test:* This study used spatial autocorrelation to measure the similarity or dissimilarity of a spatially mapped variable. Spatial autocorrelation can be positive or negative. Positive spatial autocorrelation indicates that similar values are closer together (i.e., clustered), while negative spatial autocorrelation indicates that dissimilar values are dispersed and not clustered. The global Moran’s I statistic measures the overall spatial autocorrelation of a variable throughout a study area. This study used this statistic to assess whether VL occurrence (i.e., positive and negative cases) exhibited spatial clustering or dispersion.

*Collinearity test:* The correlation coefficient (r) between the variables was also calculated to reduce the redundancy problem that could arise due to collinearity. Redundancy makes it challenging for the model to isolate the individual effect of each predictor, leading to unstable coefficient estimates, and, in some cases, the “not available” (NA) error occurs. Furthermore, collinearity in variables presents a challenge to model interpretability, especially when trying to determine the importance of each correlated variable. In this study, one of the two highly collinear variables with an r of ±0.7 was dropped from the study.

Considering the assumption of spatial autocorrelation and collinearity test, the following statistical models were selected:

i. *Spatial logistic regression:* The logistic regression model calculates the relationship between the independent variables and the probability of a categorical outcome by transforming the odds into logarithmic odds. Coefficients are estimated using maximum likelihood estimation, which iteratively identifies the optimal fit by maximizing the log-likelihood function. Once the optimal coefficients are determined, the conditional probabilities are calculated to predict the outcome variable. Spatial logistic regression extends this approach by incorporating spatial effects through a covariance function to account for spatial dependencies (53). In this study, spatial logistic regression was used to assess the relationship between covariates and the probability of the occurrence of VL while accounting for spatial autocorrelation in the data. This was implemented in the spaMM package version 4.5.0 in the R statistical software. The spatial logistic equation is defined in Equation 7; where $\log\left(\frac{P(Y_{i}=1)}{1-P(Y_{i}=1)}\right)$ is the logit (log-odds) of the binary outcome, $\beta_{1},\ldots ,\beta_{k}$ are fixed effect coefficients, $\beta_{0}$ is the intercept, $x_{1i},\ldots ,x_{\mathrm{ki}}$ are the independent variables (predictors) for the observation i, $\gamma_{i}$ is the random effect for the monthly variation, $\beta_{1},\ldots ,\beta_{k}$ are the coefficients for the corresponding predictors, and $S(x_{i})$ the spatial random effect which captures the spatial correlation in the data.
  (7) $$\log\left(\frac{P(Y_{i}=1)}{1-P(Y_{i}=1)}\right)=\beta_{0}+\beta_{1}x_{1i}+\beta_{2}x_{2i}+\cdots +\beta_{k}x_{\mathrm{ki}}+\gamma_{i}+S(x_{i})$$
ii. *Bayesian spatial model:* The Bayesian spatial model integrates spatial correlation into data analysis across geographical regions, making it particularly effective for data exhibiting spatial dependence, where nearby locations share similar outcomes due to unobserved factors. Bayesian inference estimates posterior distributions by updating prior distributions with observed data. While Markov Chain Monte Carlo (MCMC) methods have traditionally been used for this purpose, they can be computationally intensive, especially in high-dimensional parameter spaces like spatial data analysis, due to long burn-in periods and the need for subsampling to ensure convergence. More efficient methods, such as integrated nested Laplace approximations (INLA) and stochastic partial differential equation (SPDE) approaches, have emerged to address these limitations. INLA, in particular, provides a computationally efficient alternative for latent Gaussian models, improving the feasibility and speed of model fitting in complex high-dimensional analyses (54). The INLA and SPDE approaches were implemented in the R-INLA package version 24.06.27 in R statistical software. The Bayesian spatial model is defined in Equation 8; where $y_{i}$ is the response variable for the i-th observation, $\beta_{0}$ is the intercept, $\sum_{m=1}^{M}[\beta_{m}X_{m,i}]$ represents the sum of covariates $X_{m,i}$ multiplied by their respective coefficients $\beta_{m}$, $S(x_{i})$ is the spatial random effect associated with location $s_{i}$, and $\gamma_{i}$ is the random effect for the monthly variation.
  (8) $$y_{i}=\beta_{0}+\sum_{m=1}^{M}\beta_{m}X_{m,i}+S(x_{i})+\gamma_{i}$$

### 2.5 Performance evaluation and model tuning

At different lags, the performance of the models (statistical and machine learning) was evaluated using the accuracy, precision, recall, F1 score, and the area under the curve of the receiver operating characteristic curve (AUC-ROC). Accuracy was measured by the proportion of correctly classified VL cases out of all cases. Precision measured how many of the predicted positive VL cases were positive. Recall measured how well the model identified actual positive VL cases. F1-score was the harmonic mean of precision and recall, which balanced the trade-off between false positives and false negatives in VL cases. The AUC curve evaluated the model’s ability to distinguish between classes by plotting the true positive rate (Recall) against the false positive rate (FPR) at various threshold values, with the AUC representing the model’s overall discrimination power. The FPR was the proportion of all actual negative VL cases that were incorrectly classified as positive VL cases.

The AUC metric is widely considered a more robust and reliable evaluation metric compared to accuracy, recall, precision, and F1-score. AUC evaluates the discriminative ability of the model across all possible thresholds, accounting for true positive and false positive rates (55, 56). Moreover, AUC incorporates both sensitivity and specificity across multiple thresholds, offering a more comprehensive assessment (57). AUC is advantageous compared to the other metrics (accuracy, recall, precision, and F1-score) in that it is threshold independent, robust to class imbalance, and capable of revealing subtle but statistically significant differences when comparing the performance of various models that other metrics may overlook (55, 56). Therefore, this research relied on AUC as the primary performance evaluation metric.

The statistical models we tuned while setting them up, as stated in Section 2.4.2. For the case of machine learning models (discussed in Section 2.4.1), their hyper-parameters were tuned using the grid search method (from the Scikit-learn library). The grid search was implemented to perform an exhaustive search and identify the possible combination of hyper-parameters that gave the best AUC score from the pre-defined list of hype-parameter ranges defined in Table 2.

**Table 2:** Hyper-parameters search ranges of the different machine learning models implemented.

Machine classifier	Parameter	Grid search range
Random forest	The number of trees (n_estimators)	[10, 20, 40, 80, 160, 320, 640]
	The maximum depth of the tree (max_depth)	[None, 5, 10, 15, 20]
Support vector classifier	Regularization parameter (C)	[0.5, 1, 1.5]
	Kernel type	[radial basis function, sigmoid]
AdaBoost	The number of trees (n_estimators)	[10, 20, 40, 80, 160, 320, 640]
	learning_rate	[0.1, 0.2, 0.3, 0.4, 0.5]
Logistic regression	Inverse of regularization strength (C)	[0.5, 1, 1.5]
Extra trees	The number of trees (n_estimators)	[10, 20, 40, 80, 160, 320, 640]
	The maximum depth of the tree (max_depth)	[None, 5, 10, 15, 20]

**Figure 5:**
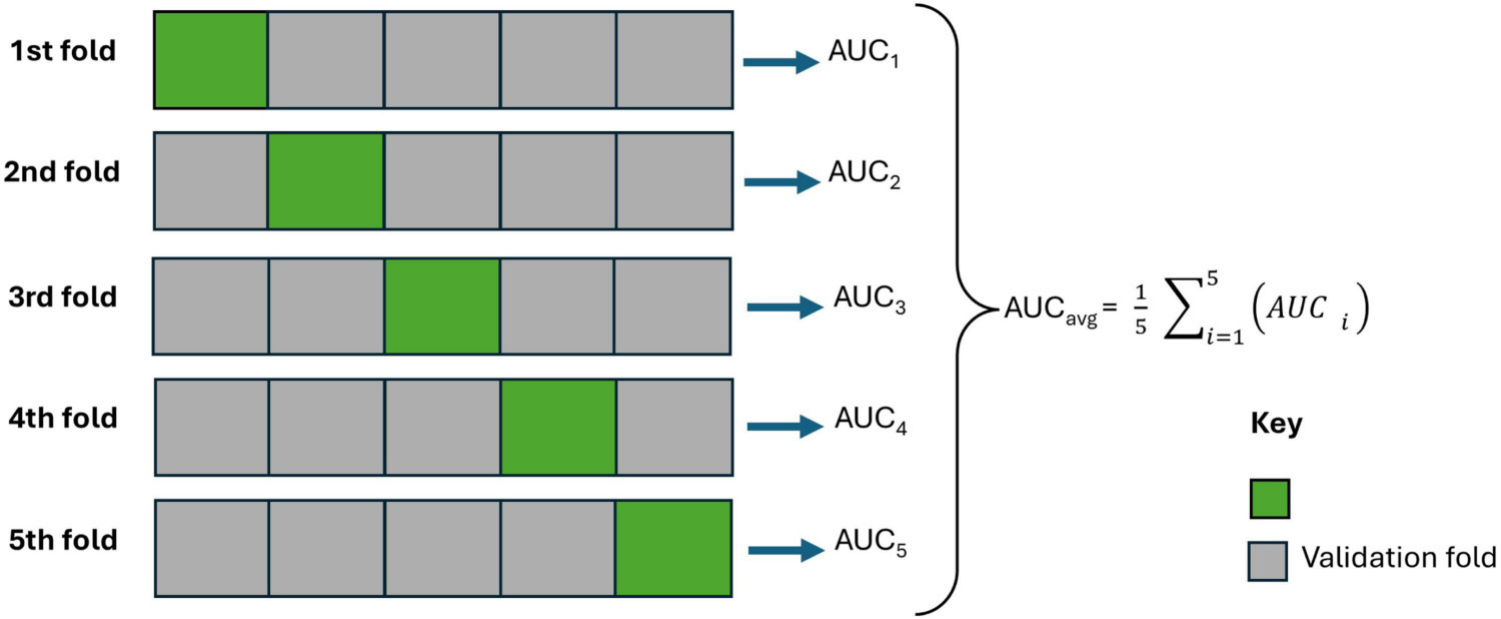
An illustration of the cross-validation strategy with 5 folds while being evaluated using area under the curve performance metrics.

Thereafter, all (statistical and machine learning) models were rigorously tested on unseen datasets using cross-validation (from the Scikit-learn library). In the cross-validation implementation, the data was divided 5 times (that is, k=5 folds) without shuffling; the model was trained with one fold and evaluated with the remaining unseen folds (validation). As shown in Figure 5, each model was trained using k−1 of the five folds as training data, and then the resulting model was validated on the remaining (unseen) part of the dataset that was used as a test set, where the AUC was calculated to measure the performance of the model. Subsequently, the AUC scores were averaged. Lastly, for each model, the highest AUC score was recorded across the lagged datasets, and the subsequent lagged month value (0 to 8) was also recorded. The model with the highest AUC value across all models was selected to generate the epidemiological risk map; Section 2.6. Afterward, important features were drawn and discussed; Section 2.7. The less competitive models were ignored.

### 2.6 Risk map generation

This research generated the December 2024 epidemiological risk map using the model that performed best. Taking into account the lag in the dataset (created in Section 2.2) that gave the best performance, the best performing model (from those trained in Section 2.4) generated the (positive) probability output of each observation (in view of some of the variables in Table 1) in the month before (considering the optimal value of the lag month) December 2024. The probabilistic outputs (predictions) were associated with the geo-location of the record. This provided a future (considering the identified lag month of the model) assessment of a possible VL risk across the Turkana region. The future predictions, along with their geo-locations, were stored and used to create a continuous spatial map using the inverse distance weighting (idw) interpolation function. The Turkana shapefile was also loaded into the R or Python programming environment to define the area of interest. Using this shape file, a resolution of 1 km was defined to create an empty raster that served as the surface of interpolation for the output. The *idw()* function was used to interpolate the infection probabilities to make them continuous on the raster grid. The resulting interpolated grid raster was then saved as a Tag Image File Format (TIFF) and then loaded into the Quantum Geographic Information System (QGIS) to generate the lagged risk map for December 2024.

### 2.7 Explainability and interpretation of the model

The SHapley Additive exPlanations (SHAP) were used to interpret the performance of the best model. Based on game theory, SHAP quantifies the contribution of each feature to the final prediction, which is similar to how the impact of a player is assessed in a cooperative game (58). In this study, considering the best-performing model, SHAP values were used to explain how individual features influenced the outcome (predicted) variables. This interpreted and explained the relative importance of different features and revealed the magnitude/interactions of their values to inform the outcome/response variable.

## 3 Results

### 3.1 Performance scores

#### 3.1.1 Statistical models

i. *Spatial autocorrelation score:* The global Moran’s I statistics indicate positive spatial autocorrelation for infections across the study area as well as in the residuals of the logistic model. The global Moran’s I for infections gave a statistic of 0.0710, and a *P*-value of 0.0. In contrast, the global Moran’s I test applied to the residuals of the logistic regression produced a statistic of 0.0572 and a *P*-value of 0.0007.
ii. *Collinearity scores:* There was a high correlation (*r* ± 0.7) among maximum temperature, mean temperature, minimum temperature, and the elevation. The maximum temperature was retained, and the others were excluded from the analysis. Therefore, the variable fed into the spatial models was infections (i.e., both positive and negative) as the outcome/target/dependent variable, and the independent/feature variables were sex, age groups, proximity to healthcare, population density, canopy height, maximum temperature, mean humidity, total precipitation, and distance to water bodies. Spatial autocorrelation was factored into the models by adding a spatial covariate function. The month of infection was also included in the models as a random effect to account for the monthly variability of the disease.
iii. *Evaluation scores:* The Bayesian spatial model and the spatial logistic model were trained and tested, and the results were recorded as shown in Table 3.

**Table 3:** Area under the curve and confusion metrics percentage performance metrics scores of statistical and machine learning models.

Category	Model	ROC percentage score		Confusion matrix percentage scores				
		AUC	Best lag	Accuracy	Precision	Recall	F1-score	Best lag
(a) Statistical models	(i) Bayesian Spatial	67.3	5	60.6	67.7	29.9	41.3	8
	(ii) Spatial logistic	68.4	5	64.4	63.3	57.0	59.8	5
(b) Machine learning	(i) Random forest	66.6	3	63.9	64.4	63.9	63.5	4
	(ii) Support vector classifier	68.7	3	64.0	65.3	64.0	62.9	4
	(iii) **AdaBoost**	**71.2**	**3**	67.0	67.4	67.0	66.7	4
	(iv) Logistic regression	66.6	3	63.6	64.5	63.6	62.7	4
	(v) Extra trees	70.1	3	**67.4**	**68.3**	**67.4**	**66.9**	4

In bold is the best performing model and the best performance metrics values of respective models.

The Bayesian spatial model and the spatial logistic regression model recorded an AUC of 67.3% and 68.4%, respectively. Moreover, the Bayesian spatial model recorded an accuracy, precision, recall, and F1-score of 60.6%, 67.7%, 29.9%, and 41.3%. The spatial logistic regression recorded an accuracy, precision, recall, and F1-score of 64.4%, 63.3%, 57.0%, and 59.8%. Both models recorded their highest AUC scores at lag 5. Considering AUC, the spatial logistic model performed better compared to the Bayesian spatial model. The same model also performed considerably well on accuracy, recall, and F1-score.

#### 3.1.2 Machine learning models

The performance of the five machine learning models is presented in Table 3. Considering AUC, all the models recorded their highest scores at lag 3. From the five models, the AdaBoost model recorded the best AUC score of 71.2%. The AdaBoost accuracy, precision, recall, and F1-score deviated minimally compared to the extra trees, which had the highest scores. Generally, across the statistical and machine learning models, the AdaBoost algorithm gave the highest AUC performance score. The algorithm was then used to generate epidemiological risk maps as explained in the following section. The other models were ignored, since they recorded a relatively low AUC performance.

### 3.2 Risk map generation and ground truthing—the AdaBoost model

**Figure 6:**
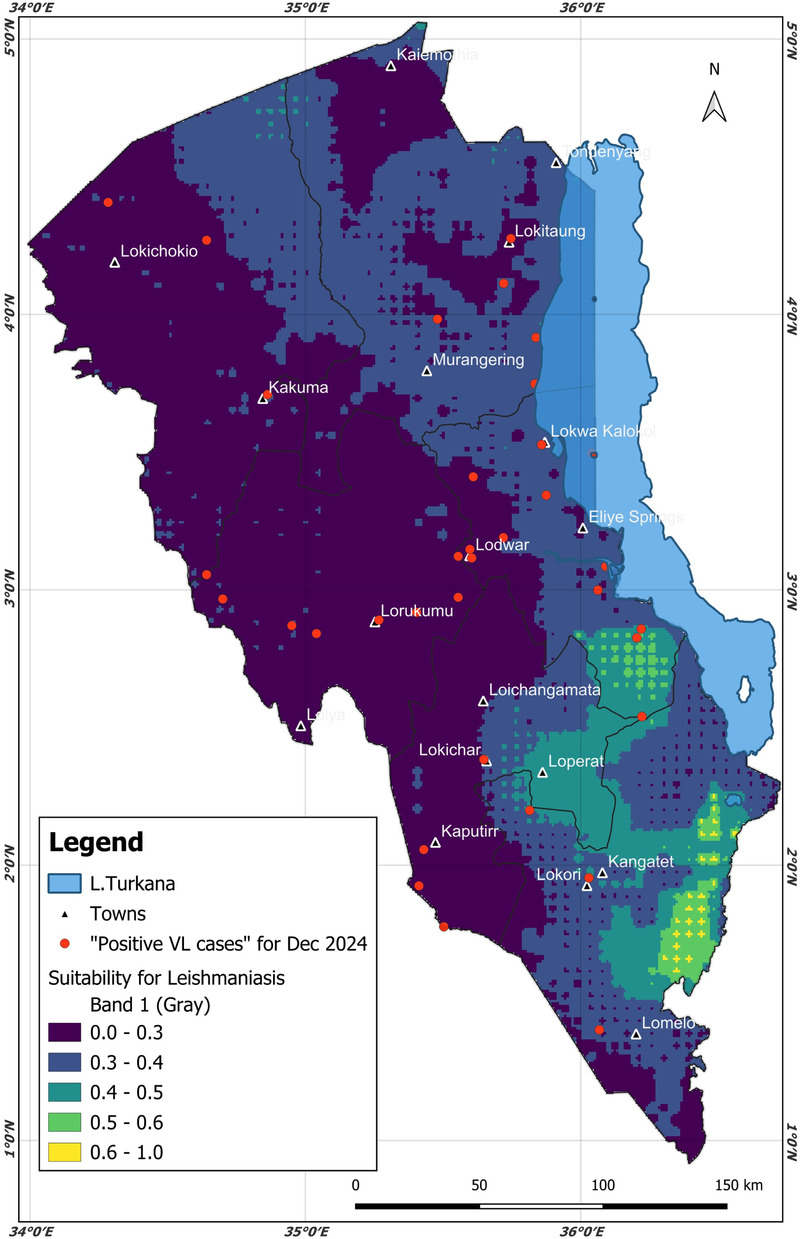
The predicted December 2024 Turkana County. Different colors on the map highlight different types of risk for the areas. The December 2024 visceral leishmaniasis cases from the same region were overlaid (the red points/markers) to ground truth the model. Map from: https://dudumapper.icipe.org/, licensed under MIT License.

As discussed in the previous section, ideally, the AdaBoost machine learning model identified an optimal lag (incubation period) of 3 months to predict the surge (increase in) VL cases in hospitals in Turkana County. The 3-month period could be the optimal lead time for decision-making and preparations against VL. To predict future scenarios with the identified 3-month lead time, this research generated a December 2024 VL epidemiological risk map, shown in Figure 6. Taking into account the optimal 3-month lag prior to December 2024, the AdaBoost model was ingested with variables in October 2024 (mainly environmental and demographic), namely, minimum temperature, elevation, greenness, wetness, brightness, soil type, mean temperature, maximum temperature, population density, LULC, forest height, distance from water bodies, humidity, and precipitation; the data were collected and cleaned using the data protocol described in Sections 2.2, 2.3. Patient data (mainly age and sex) were not considered, since realistically this cannot be known in the future. The risk map was deployed on a web application (https://dudumapper.icipe.org/). We note that the December 2024 positive VL cases from the 12 treatment centers in Turkana County were overlayed on the map to ground-truth (validate) the model. The model identified some points in the relative mid- to high-risk zone, above the 0.4 suitability bands.

### 3.3 Explainability and interpretation—the AdaBoost model

**Figure 7:**
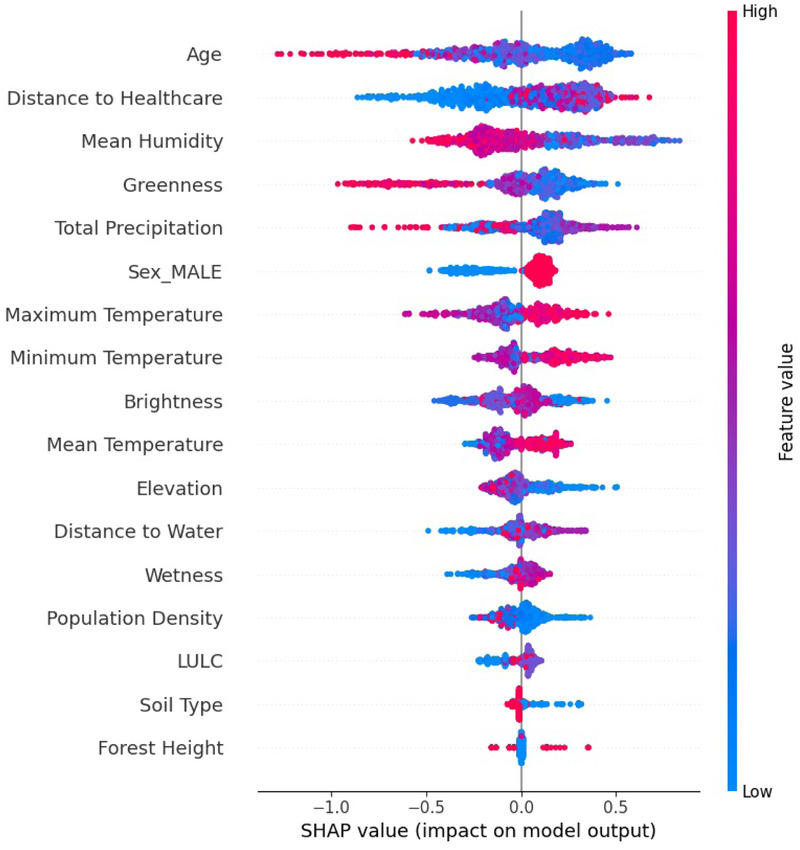
The AdaBoost summary plot showing the order of variable importance and directionality.

The SHAP summary plot, in Figure 7, provides a detailed instance-level interpretation of how different features influenced the occurrence of VL. The red or blue points in the plot represent atomic observations/values. The color gradient, which varies from blue to red, represents the actual values of each feature. Blue signifies low feature values, while red represents high values. The *x*-axis shows the SHAP value, which indicates the impact of a given feature on the model’s output. A positive SHAP value means that the feature increases the likelihood of VL occurrence, whereas a negative SHAP value suggests a reduced likelihood. The *y*-axis lists the features in descending order of importance, which implies that the features at the top had the greatest influence on the model predictions and were equally of greater importance as VL drivers. A SHAP summary plot of the AdaBoost model was generated as shown in Figure 7. In descending order, the AdaBoost model informs us that the following variables (top 9) were important (significant drivers) for VL, namely, age (mixture of positive and negative SHAP values), distance to healthcare (negative SHAP values), mean humidity (mixture of positive and negative SHAP values), greenness (positive SHAP values), total precipitation (negative SHAP values), sex (male) (positive SHAP values), maximum temperature (negative SHAP values), minimum temperature (negative SHAP values), and brightness (positive SHAP values).

**Figure 8:**
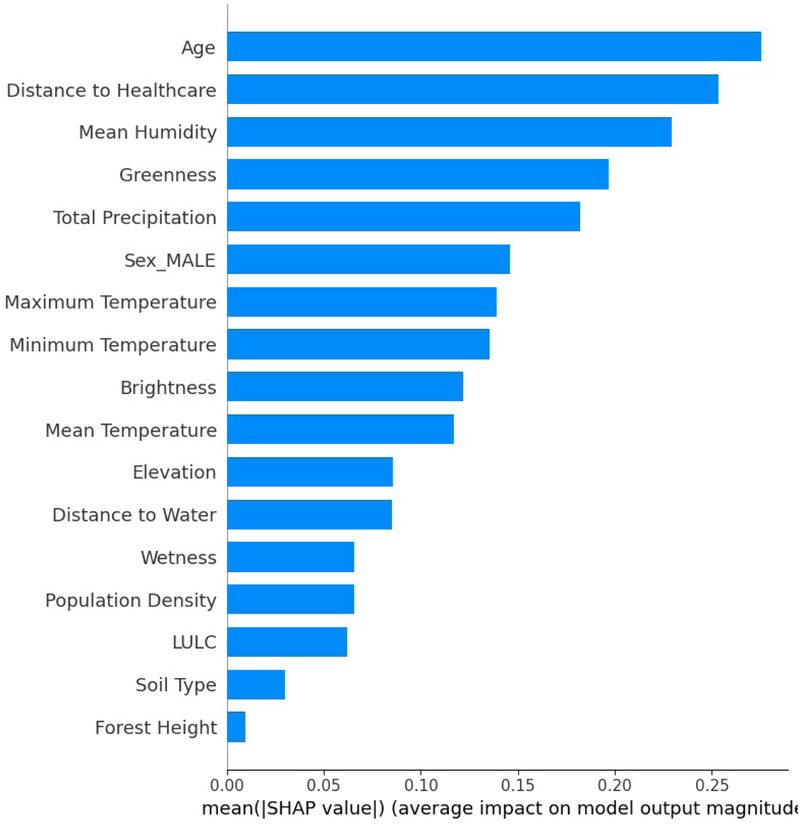
The AdaBoost feature importance summary and the contribution of each variable to the model.

The SHAP feature importance plot, in Figure 8, illustrates a global assessment of the relative contribution of each feature to the model output. In contrast to the SHAP summary plot, which illustrates the impact of feature values at the individual observation level, this bar plot aggregates the absolute SHAP values across all observations and ranks features based on their overall influence. The *x*-axis represents the mean SHAP value, quantifying the average contribution of each feature to the occurrence of VL, while the *y*-axis displays the features in descending order of importance.

## 4 Discussion

Lodwar County Referral Hospital recorded the highest number of positive VL cases. It is the largest hospital in Turkana County and experiences an influx of patients from other hospitals in the county (59). The WHO strategic framework for the elimination of VL, (3), notes that Turkana County experiences many transnational cases of VL that are diagnosed and treated in Turkana hospitals. For instance, Namoruputh PAG Health Centre, Lopiding Sub-County Hospital, Kakuma Mission Hospitals, and Kaikor Sub-County Hospital treat patients from Uganda, South Sudan, and Ethiopia, respectively.

The AUC performance metric was used to test a model’s ability to discriminate positive compared to negative cases, which was the main aim of this research. Therefore, considering the AUC, the AdaBoost was the best-performing model. The model was then used to generate the future epidemiological risk of VL in Turkana County. The other models were ignored, as they provided lower AUC scores. The AdaBoost model identified a 3-month lag (incubation) period, that is, the time a patient could fall sick and go to the hospital after being infected with VL. The 3-month period is within the disease incubation period (0 to 8 months) as identified by (39, 40). Based on weather and ecological variables for October 2024, the model generated the epidemiological risk map for December 2024. The resulting risk map can be accessed on the interactive DuduMapper platform (https://dudumapper.icipe.org/). The map illustrates the epidemiological risk levels predicted for VL in Turkana County for December 2024. In the map, areas with a predicted probability greater than 0.4 were classified as mid- to high-risk zones for VL cases. However, the other regions show potential for VL surge, though at a minimal level. Notably, the AdaBoost model identified several such regions, including areas adjacent to Lake Turkana, the Kerio Delta region, sections in Lokori in Turkana East, and some parts of north Turkana West. These identified areas align with environmental and socio-ecological conditions conducive to VL transmission. As highlighted by (60–64), proximity to water bodies supports socio-economic activities such as bathing, fishing, and irrigation, which increase human-vector contact. In addition, such environments maintain soil moisture and provide resting habitats essential for the development and survival of sandflies, the primary vectors of VL.

SHAP analysis drawn by the AdaBoost model trained on the lag 3 dataset indicated that the most important variables in descending order were age, distance to healthcare, mean humidity, greenness, total precipitation, sex (male), maximum temperature, minimum temperature, and brightness. Maximum and minimum temperatures were associated with an increase in infections in Turkana County. Temperature is crucial in the transmission dynamics of VL, as it reduces the time required for vector development while enhancing biting rate, vector capacity, and parasite replication within the vector (65). Previous studies carried out in India, Greece, China, Ethiopia, Sudan, and Brazil have shown a positive association between temperature and VL transmission (9, 11, 66, 67). In the Gangetic Plain, India, a temperature range from 25 ${}^{\circ }$C and 27 ${}^{\circ }$C was found to be the most ideal for VL transmission (66). In Kashi prefecture, China temperature ranges between $21{\;}^{\circ }$C and $28{\;}^{\circ }$C, positively associated with VL (9). In Ethiopia, annual temperatures ranging from $20{\;}^{\circ }$C and $37{\;}^{\circ }$C were found to be a significant predictor of VL in the country (67).

In Southeast Asia, a range of temperatures between 15 and 38 degrees Celsius was found to alter vegetation and human-vector interaction, thus influencing the spread of disease in the area (68).

Total precipitation has a significant effect on the presence and absence of VL vectors that transmit visceral leishmaniasis. Several studies have documented the mixed effects caused by total precipitation (15, 65, 69, 70). On the other hand, total precipitation can create ideal soil moisture and therefore facilitate an ideal breeding environment for sandflies (15, 65). The aftermath of rain is associated with the growth of woody plants over time (including fast-growing invasive woody species) along swamps and seasonal riverbeds. These plants often provide shade for herders during the dry season. Plants also cause fissures and crevices that form humid environments in which sandflies can hide and also breed (15). Consequently, there is an increased interaction between humans and sandflies along riverbeds and swamps, increasing the risk of VL transmission. Too much rainfall is also associated with vector expansion in new areas, as seasonal water paths can carry sandfly larvae from endemic areas to non-endemic areas (15). In Brazil, heavy rain has been associated with flooding, sewage overflows, and trash accumulation in urban areas (70). These poor sanitary conditions provide sandflies with the necessary nutrients for larval development and therefore have been associated with VL outbreaks in urban areas (70). However, too much rain can destroy immature eggs from sandflies and disrupt the life cycle of the vector, flying, and resting capacity, all of which can lead to a reduction in VL transmission (65, 69).

Approximately 75% of all cases occur in individuals under 19 years of age, suggesting important implications for vector behavior and transmission dynamics. These findings align with previous research, which consistently reports that VL affects primarily younger populations (20, 71–73). In 2022, the WHO highlighted that 66% of all VL cases are concentrated in eastern Africa, half of which were observed in children under 15 years of age. A study carried out in the Amhara region in Ethiopia found that children under 15 years of age were 3.3 times more likely to be infected with the disease compared to adults (74). Factors contributing to VL infection among children can vary between different societies. However, immature immune response and malnutrition are some of the key factors driving VL infections among children (20, 71–73). In some communities, such as Ethiopia, children are responsible for herding, which predisposes them to areas that are vector-infested, increasing their exposure to VL (71). Children playing outside, especially in areas endemic to VL, are also at increased risk of getting infected. In addition, children who live with large families also have an increased risk of infection (21).

In this study, the male gender was an important feature that increased the likelihood of VL infection. Previous studies show that being male is associated with an increased risk of infection (21, 74–76). A study in Ethiopia pointed out that males were 67% more likely to be infected with VL than females (75). Another study in Amhara, Ethiopia, established that males were 4.6 times more likely to be infected with the disease compared to females (74). A study conducted in India and Nepal determined that men were 2.4 times more likely to have VL compared to women. The increased risk for men can be attributed to outdoor activities such as herding, farming, and sleeping outside, increasing their risk of exposure to sandfly bites (21). Furthermore, volatile profiles of males and females need further elucidation as a possible explanation for the attraction of sandflies to a blood meal varies between sexes (77).

Relative humidity plays an important role in the multiplication of the leishmania parasite in sandflies. It influences the development of larvae, the gonotrophic cycle, the longevity, and the duration of the extrinsic cycle of sandflies (78). In addition, relative humidity also influences the development and survival of eggs until the dormancy stage of the sandflies (79, 80). How relative humidity affects VL transmission is dependent on geographic location and its relationship with rainfall and temperature. This is because the combined effects of rainfall, evaporation, and temperature regulate ambient air humidity, which in turn influences the survival and activity of sandflies (78). Valero et al. (81) in São Paulo, Brazil, determined that relative humidity was dependent on the amount of precipitation, and an increase in the amount of rain led to an increase in relative humidity and subsequently in vector abundance and VL occurrence. Studies by (9) established that relative humidity was associated with temperature. An increase in temperature was associated with a decrease in relative humidity and ultimately an increase in the number of VL occurrences.

From an Earth observation perspective, the greenness component of the tasseled cap transformation in remote sensing is associated with low to high values of the greenness of vegetation, while the brightness component is associated with providing insights into bare/partially covered soil, man-made and natural features such as asphalt, concrete, rock outcrops, gravel, and other bare areas (82). The SHAP results in this investigation indicated low values of greenness (low density of vegetation) and moderate brightness (bare soils). This is a characteristic of semi-arid regions such as Turkana County. Martín et al. (83) noted that vegetation (such as tree cover, grassland, and scrubland) provides a conducive habitat for vectors and rodent hosts. Scrublands and grasslands provide a conducive environment for sandflies due to a combination of vector ecological factors. Areas with vegetation coverage, such as those with herbaceous plants and shrubs, offer favorable conditions for the development of sandflies. These environments tend to accumulate organic matter and litter, providing breeding sites for immature sandflies, while nectar sources support adult sandflies. In addition, acacia trees offer an attractive food source (84) and a resting place (85) for sandflies. Grassland vegetation influences human herding patterns and movement, which can further increase the risk of exposure (7).

World Health Organization (3) noted that access to VL diagnosis and treatment centers is challenging, such that patients have to travel a considerable distance to access a VL diagnosis and treatment center, and some patients may die of VL complications before getting to the healthcare facilities. Turkana County has a vast semi-arid land mass. The region is marginalized, underserved, and has poor road infrastructure (34). Ideally, the diagnosis and treatment centers in that region are not sufficient to serve the population. This leads to insufficient access to VL health care. Patients infected with VL travel a long distance to seek treatment. In the presence of a female *Phlebotomus* sandfly, sick patients pose a risk of transmission of the disease in the population.

## 5 Conclusion

Epidemiological, ecological, and environmental factors are multi-factorial risk drivers in the transmission dynamics of visceral leishmaniasis. Understanding the major determinants, the incubation period, and areas of high/low risk can be valuable to stakeholders in effective disease prevention and control. In this research, the AdaBoost machine learning classifier emerged as the best-performing model. It identified a lag time of 3 months between patient infection and when they seek treatment. The model also identified mean temperature, total precipitation, distance to healthcare, age, mean humidity, sex (male), and land use land cover (LULC) as the most influential predictors of visceral leishmaniasis (VL) transmission. A future epidemiological map (that is, October 2024) was generated from the AdaBoost model from the December 2024 weather and environmental conditions and deployed on a web application—https://dudumapper.icipe.org/. In the application, registered users receive early warning emails of possible future risks of VL. With the 3-month lead, it is anticipated that the early warning system will provide valuable insights into early preparedness. These approaches provide an intelligent and resource-effective route to identify the disease incubation period and high-risk areas and implement specific interventions in a timely and cost-effective manner, compared to manual vector and disease surveillance strategies, mainly trapping vectors and disease diagnosis and treatment. However, future studies can consider the integration of socio-economic variables, vector data, and possibly other spatial models for long-term monitoring of VL risk factors. Also, the models and data can be optimized since we found that soil type and population density were ranked as less important; this contradicts the understanding of the domain expert and can be further investigated. However, these intelligent and dynamic approaches can provide timely and cost-effective data-driven insights to various stakeholders, such as the Kenya Ministry of Health, the International Center for Insect Physiology and Ecology, and the Kenya Medical Research Institute. These can be invaluable insights in the preparation, control, and elimination of VL in disadvantaged and marginalized rural communities in Turkana County in Kenya and beyond, and the building of resilience against VL.

## Data Availability

The raw data supporting the conclusions of this article will be made available by the authors, without undue reservation.
